# Molecular Mechanisms and Therapeutic Implications of Endothelial Dysfunction in Patients with Heart Failure

**DOI:** 10.3390/ijms24054321

**Published:** 2023-02-21

**Authors:** Vasiliki Tsigkou, Evangelos Oikonomou, Artemis Anastasiou, Stamatios Lampsas, George E. Zakynthinos, Konstantinos Kalogeras, Maria Katsioupa, Maria Kapsali, Islam Kourampi, Theodoros Pesiridis, Georgios Marinos, Michael-Andrew Vavuranakis, Dimitris Tousoulis, Manolis Vavuranakis, Gerasimos Siasos

**Affiliations:** 1 3rd Department of Cardiology, Medical School, National and Kapodistrian University of Athens, Sotiria Chest Disease Hospital, 11527 Athens, Greece; 2 1st Department of Cardiology, Medical School, National and Kapodistrian University of Athens, Hippokration General Hospital, 11527 Athens, Greece; 3 Cardiovascular Division, Brigham and Women’s Hospital, Harvard Medical School, Boston, MA 02115, USA

**Keywords:** heart failure, endothelial dysfunction, pathophysiology, molecular mechanisms

## Abstract

Heart failure is a complex medical syndrome that is attributed to a number of risk factors; nevertheless, its clinical presentation is quite similar among the different etiologies. Heart failure displays a rapidly increasing prevalence due to the aging of the population and the success of medical treatment and devices. The pathophysiology of heart failure comprises several mechanisms, such as activation of neurohormonal systems, oxidative stress, dysfunctional calcium handling, impaired energy utilization, mitochondrial dysfunction, and inflammation, which are also implicated in the development of endothelial dysfunction. Heart failure with reduced ejection fraction is usually the result of myocardial loss, which progressively ends in myocardial remodeling. On the other hand, heart failure with preserved ejection fraction is common in patients with comorbidities such as diabetes mellitus, obesity, and hypertension, which trigger the creation of a micro-environment of chronic, ongoing inflammation. Interestingly, endothelial dysfunction of both peripheral vessels and coronary epicardial vessels and microcirculation is a common characteristic of both categories of heart failure and has been associated with worse cardiovascular outcomes. Indeed, exercise training and several heart failure drug categories display favorable effects against endothelial dysfunction apart from their established direct myocardial benefit.

## 1. Introduction

Heart failure (HF) is a heterogenous clinical syndrome with a broad range of symptoms and signs, which are attributed to divergent underlying etiologies that induce structural or functional abnormality of the heart [1]. HF affects more than 60 million adults globally and is characterized by severe morbidity, mortality, and poor quality of life [1]. Despite the decreased age and sex-adjusted incidence for both heart failure with reduced ejection fraction (HFrEF) and heart failure with preserved ejection fraction (HFpEF), the prevalence remains high and is projected to increase worldwide due to the aging process, improved therapeutic options for ischemic heart disease, and the availability of effective evidence-based therapies [2]. Although the prognosis of HF has, in general, been improved, it should be emphasized that mortality remains high according to recent studies, irrespectively of left ventricular ejection fraction (LVEF) and type of HF (acute or chronic) [2].

HFrEF is mainly attributed to the loss of cardiomyocytes due to ischemia, myocarditis, or genetic mutations, which trigger the mechanisms of cardiovascular remodeling [3]. Patients with HFrEF are predominantly men, usually post-myocardial infarction (MI), and develop more frequently a profile of eccentric hypertrophy of the left ventricle (LV) due to pressure overload [3]. On the other hand, HFpEF, which is now considered to be the most common category of HF, is associated with the presence of comorbidities, such as arterial hypertension, diabetes mellitus, renal dysfunction, obesity, and increased age [4].

Endothelial dysfunction (ED) has an important role in the pathophysiology of HF since repeated episodes of microvascular dysfunction might precipitate myocardial stunning and ventricular remodeling and might be associated with HF hospitalizations [5]. Moreover, ED has been linked to the presence of a procoagulant state, the expression of adhesion molecules, and a pro-inflammatory status [6,7]. Additionally, the pathophysiology of HFpEF is quite different from HFrEF according to the literature since it is largely attributed to the existence of coronary microvascular ED due to the presence of cardiovascular risk factors, which trigger a state of systemic inflammation [8]. However, HFpEF exhibits a profile of concentric hypertrophy [3], and these differences in risk factors, as well as in the phenotypic presentation of different HF categories, may be caused by differences in molecellular mechanisms and expression [9].

Therefore, in this article, we review the molecular mechanisms underlying the association of ED with the development and progression of HF as well as the therapeutic implications of ED improvement in patients with HF.

## 2. Etiopathogenesis and Molecular Mechanisms of Heart Failure

### 2.1. Neurohormonal System Activation

Activation of neurohormonal systems is a key component of HF [10]. The sympathetic nervous system (SNS) and the renin–angiotensin–aldosterone system (RAAS) are the most important neurohormonal systems in the pathophysiology of HF and an important target of several therapeutic regimens [11]. Persistent overstimulation of SNS has been linked to the development of cardiomyocyte hypertrophy, interstitial fibrosis, inflammation, and oxidative stress, which progressively result in loss of myocardial contractility and LVEF deterioration [12]. Moreover, there is an interrelation between the RAAS system and SNS, which exacerbates cardiovascular damage [13]. It should be highlighted that aldosterone is associated with the development of ED, inflammation, and production of reactive oxygen species (ROS), which further deteriorate cardiovascular function [14,15]. All these effects are mainly driven by angiotensin (AT) receptors AT-1 since AT-2 receptors have displayed antifibrotic, anti-inflammatory, and anti-apoptotic actions due to activation of bradykinin and nitric oxide (NO) synthesis [16].

### 2.2. Oxidative Stress

Oxidative stress results as an imbalance between ROS production and their reversal by the antioxidant systems of the body; normally, a small amount of ROS is formed during mitochondrial respiration, which is detoxified by cells’ antioxidant enzymes [17]. Nevertheless, excessive ROS production at mitochondria and the presence of dysfunctional antioxidant systems are the key contributors to oxidative stress in HF [17]. ROS overproduction induces ED due to NOS uncoupling and concomitant superoxide anion and peroxynitrite release, which decreases nitric oxide (NO) availability and further causes vasoconstriction [18]. Ischemia or hypoxia further exacerbates ROS production by mitochondria which accelerates myocardial damage, both at the stages of ischemia–reperfusion injury as well as in chronic ischemic conditions [19]. ROS induces post-translational modification of cellular compartments, reversibly or irreversibly, in a process that ends in cardiac hypertrophy [20]. Indeed, protein kinase C and mitogen-activated protein kinases (MAPK) induce myocardial remodeling and hypertrophy [21]. Moreover, excessive ROS production might damage mitochondrial DNA, which has a low capacity for repair; therefore, a vicious cycle of ROS overproduction and subsequent myocardial damage occurs [22]. Indeed, oxidative stress upon neurohormonal activation deteriorates the proper mitochondrial function of cardiomyocytes; mitochondrial dysfunction causes further deficiency of mitochondrial energetics [23]. Contrarily, cardiovascular risk factors in HFpEF create an environment of oxidative stress, inflammation, and microvascular ED; nonetheless, less is understood so far about the implication of mitochondrial dysfunction and cardiac energetics and should be further investigated [23,24,25]. Importantly, oxidative stress and chronic, low-grade inflammation are important contributors to the poor regenerative properties of cardiomyocytes [26].

### 2.3. Calcium Regulation

High cytosolic calcium input in HF is particularly attributed to the dysfunction of the L-type calcium channel (LTCC); indeed, increased phosphorylation of LTCC is evident in HF and results in a compensatory leak of sarcoplasmic reticulum (SR) calcium through ryanodine receptor 2 (RyR2), which is in the uncoupled form due to chronic SNS stimulation and oxidative stress [27,28]. On the other hand, dysfunction of the sodium–calcium exchanger (NCX), which controls cytosolic calcium outflux due to the diminished transmembrane sodium gradient, results in sarcolemmal depolarization [29,30,31]. It should be mentioned that myocardial hypertrophy per se is associated with prolonged duration of isometric contraction and relaxation and molecular changes due to fibrosis and dysregulated creatine kinase system [32]. Furthermore, the transverse tubular system and proteins of the excitation–contraction coupling system display dysfunction in the aged cardiomyocytes, especially in the HF condition [33]. On the other hand, improper function and diminuted expression of cardiac SR calcium ATPase (SERCA2 a) end in reduced calcium transfer to SR [34]. Additionally, dephosphorylazation of phospholamban from protein phosphatase-1 has also been identified in HF [35]. SERCAa is controlled by hormones, microRNAs (miR), and endogenous protein inhibitors and undergoes post-translational modifications [36]. Lastly, recent data indicate that inflammation and, specifically, damage-associated molecular patterns (DAMPs, i.e., destroyed or stressed cardiomyocytes) release mediators that trigger the immune response; as a result, dysfunctional cardiac contraction and electromechanical uncoupling occur due to the unfavorable calcium homeostasis [36].

### 2.4. Impaired Metabolism and Mitochondrial Energetics

Myocardial contraction and relaxation depend on the proper energy production by cardiomyocytes, which is orchestrated by cardiac mitochondria during oxidative metabolism in the mitochondrial matrix in a process known as mechano-energetic coupling [37]. Normally, cardiac mitochondria, which are the main source of energy, ROS production, and calcium ion control, utilize free fatty acids in order to produce energy in the form of adenosine triphosphate (ATP) [37]. As a matter of fact, utilization of free fatty acids results in more efficient energy production than utilization of glucose in terms of ATP production at the expense of more oxygen consumption, though [38]. According to the literature, HF is characterized by dysfunctional energy metabolism [37]. HF is perceived to be a state of ‘energy deprivation’ due to mitochondrial dysfunction, which is attributed to the altered mitochondrial structure and function [39]. Indeed, evidence from animal studies of HF has revealed that mitochondria display hyperplasia, decreased size or fragmentation, and disruption of their inner and outer membranes [39]. Several mitochondrial proteins in HF undergo post-translational modification (such as phosphorylation, acetylation, ubiquitination, conjugation of small ubiquitin-like modifier proteins, O-linked-N-acetyl-glucosamine glycosylation, proteolysis), which results in fission and fusion of mitochondria [40]. Furthermore, HF is associated with impaired mitochondrial biogenesis, dysfunctional mitochondrial DNA replication as well as mitochondrial DNA depletion [41]. Lastly, mitochondria-related genes (such as *IFIT3, XAF1, RSAD2,* and *MX1*) have displayed associations with HF and the biological processes of oxidative stress, amino-acid metabolism, and aging [42].

### 2.5. Inflammation

Inflammation and HF display a strong interrelationship; as a matter of fact, inflammation is associated with the development of molecular, cellular, and functional changes in the heart [43,44,45]. Inflammation in HFrEF is most commonly the result of cardiomyocyte injury or loss; on the other hand, cardiovascular risk factors in HFpEF create an environment of chronic, low-grade systemic, and local ongoing myocardial inflammation, which trigger myocardial damage [46]. Moreover, activation of neurohormonal mechanisms in HF, such as the RAAS system, strengthens the inflammatory response and induces immune system activation [47]. Additionally, the presence of a pro-inflammatory milieu in HF has been associated with the tendency for thromboses [48]. Furthermore, acute HF with peripheral hypoperfusion and systemic congestion favor damage to endothelial glycocalyx and cause ED [49]. Interestingly, inflammation has been linked to worse cardiovascular prognosis in patients with HF, cardiac cachexia due to muscle wasting, higher vascular resistance, and decreased functional capacity [50].

Indeed, patients with HFpEF display higher levels of inflammatory markers such as C-reactive protein (CRP), interleukin-6 (IL-6), and tumor necrosis factor-alpha (TNF-α); furthermore, CRP levels are proportional to the number of associated comorbidities [51]. Other circulating biomarkers, such as miRs, have been involved in the pathophysiology of inflammation in HF; specifically, miR-21 is highly expressed during the course of chronic HF [52]. Indeed, miR-21 has been implicated in the development of ischemia/reperfusion injury and LV remodeling [53]. As a matter of fact, several miRs are implicated in the pathophysiology of coronary artery disease, acute coronary syndromes, post-MI myocardial remodeling, and fibrosis, and they could serve as possible biomarkers for diagnosis, prognosis, and treatment [53]. Most importantly, some mechanisms of their action involve detrimental effects on endothelial function, inflammation, and oxidative stress [53]. Last but not least, other non-coding RNAs have been studied intensively as possible pathophysiologic mediators not only for HF but also for several cardiovascular diseases [45].

On the other hand, in circumstances of myocardial damage, DAMPs activate the pathogen recognition receptor (RPR) pathway, which is responsible for the release of intracellular cytokines such as the receptor for advanced glycation end-products (RAGE) and cluster of differentiation-36 (CD-36) [54]. Dying cells release pro-oxidant mediators, ROS, IL-1β, myeloperoxidase (MPO), and matrix metalloproteinases (MMPs), which break down extracellular matrix (ECM) [54,55,56]. ROS provoke irreversible damage to cellular compartments and death of cardiomyocytes; also, oxidative stress is highly linked to the inflammatory response [57]. B-and T-cells participate in the inflammatory processes and cardiac remodeling; although T-lymphocytes have a protective role in cardiac remodeling post-MI, the balance between destroyed cardiomyocytes and the adaptive immune response is insufficient to prevent cardiac damage in HF [58,59,60].

Infections (i.e., viral infections) might result in the development of myocarditis and HF due to non-sterile inflammatory damage; as a matter of fact, in the acute phase, there is a loss of cardiomyocytes and myocardial infiltration with mononuclear cells [61]. Afterward, innate immune cells such as natural killer (NK) cells and macrophages hinder viral propagation until the adaptive immunity response begins; finally, antigen-specific T cells are activated, and in the subacute phase, the specific CD8+ cytotoxic T cells destroy the affected cells [61]. Last but not least, in the chronic phase, low-grade inflammation (due to the presence of infection or an inflammatory disease in general), which might last for years, triggers cardiac remodeling and augments the risk for dilated cardiomyopathy [62,63,64].

## 3. The Endothelium in Heart Failure

### 3.1. Endothelial Function in Patients with Heart Failure

The endothelium is a monolayer of cells that cover the inner surface of the vascular wall; apart from an anatomic barrier that separates blood flow from the vascular wall, endothelium serves as an important endocrine organ, which controls vascular tone, inflammation, and oxidative stress, cellular proliferation and coagulation [65]. Healthy endothelium produces NO from the enzyme endothelial nitric oxide synthase (eNOS); production of NO by eNOS is one of the most important mediators of endothelial-dependent relaxation as a response to mechanical stimuli (i.e., shear stress) or chemical substances (i.e., acetylcholine, arachidonic acid) [66]. Interestingly, eNOS is calcium-calmodulin dependent, and its activation depends on the intracellular concentration of calcium [66]. Sufficient NO production is responsible for anti-inflammatory, antioxidant, anti-coagulant, and vasodilative effects [67]. Moreover, normal endothelial function affects almost every vascular bed, such as myocardial and coronary circulation and renal, systemic, and pulmonary circulation [68].

Reduced NO production due to ED might contribute to hemodynamic compromise in the settings of acute HF [69]. According to the literature, decreased production of NO by eNOS has been linked to augmented vascular tone, cellular proliferation, and myocardial remodeling [69]. Furthermore, it has not been determined if venous congestion and fluid accumulation are the initial trigger or the result of ED in patients with acute HF [70]. Moreover, ED is also implicated in the pathophysiology of cardiorenal syndrome, which is characterized by the bidirectional dysfunction of the heart and kidney due to acute or chronic dysfunction of either organ or due to another systemic disease [48].

On the other hand, acute endotheliitis might present in acute HF and has been associated with ED and impaired NO production due to excessive oxidative stress, inflammation, and vasospasm upon an initial myocardial insult [68]. As a matter of fact, acute endothelial injury is a key characteristic of Takotsubo cardiomyopathy and is related to the presence of an excessive inflammatory response, oxidative stress, and SNS activation [71]. Experimental data in hypertensive rats demonstrate that apart from the pressure-dependent uncoupling of eNOS during pulmonary edema, disrupted endothelial cell integrity and impaired endothelial mechanotransduction favor eNOS uncoupling and excessive ROS production during episodes of acute HF [72].

Chronic HF is characterized by disturbance of normal endothelial function, which is associated with poor cardiovascular prognosis irrespectively of the cause or severity of HF [69,73,74]. Patients with chronic HF display vasoconstriction and deteriorated peripheral tissue perfusion due to ED; as a result, myocardial injury occurs, whereas poor tissue perfusion exacerbates the pre-existing vasoconstriction of both renal and coronary vascular beds [69]. Excessive oxidative stress in chronic HF is attributed to the dysfunctional antioxidant cellular defense systems due to eNOS uncoupling and impaired NO production [69]. Additionally, oxidative stress has been implicated in the dysfunctional regulation of calcium ions during systole and impaired cardiac relaxation [75]. Furthermore, oxidative modification and damage of cellular phospholipids result in high endothelial permeability and loss of endothelial integrity [76]. Decreased NO/ endothelin-1 (ET-1) ratio in chronic HF is linked to the continuous impairment of cardiac function, which is reflected by echocardiographic indices such as LVEF and LV short-axis shortening rate [77]. Lastly, increased expression of serum soluble angiotensin converting enzyme-2 (ACE) is related to poor exercise tolerance and raised asymmetric dimethyl-arginine (ADMA) levels, implying its association with the development of oxidative stress-induced ED [78].

Regarding myocardial fibrosis, the transition of endothelial cells into fibroblast-like cells in chronic HF, which is termed endothelial-to-mesenchymal transition, has been intensively investigated recently [79]. There is evidence that epigenetic control of gene transcription and translation, including DNA methylation, histone modifications, and the actions of non-coding RNAs (miR, long non-coding RNAs, and circular RNAs), are implicated in these cellular processes in chronic HF [79]. As for miR, their role in the mechanisms of vascular repair has already been established for HF [77]. Moreover, RhoA/Rho kinase overexpression is another pathway of myocardial fibrosis and LV remodeling in chronic HF, associated with impaired NO bioavailability [80]. Lastly, according to a recent study in patients with chronic HF, apoptotic endothelial cell-derived micro-vesicles such as cluster of differentiation 31 (CD31)+/annexin V+ might discriminate different HF categories along with the measurement of classic biomarkers of fibrosis such as galectin-3 [81].

On the other hand, another important pathophysiologic aspect of chronic HF is the dysfunction of endothelial circulating progenitor cells (EPCs); as a matter of fact, the pathway of AMP-activated protein kinase is involved in the regulation of EPCs number and expression and could be a possible therapeutic target for HF [73,82]. Additionally, deteriorated NO production in chronic HF exhibits detrimental effects in the expression of Vascular endothelial growth factor (VEGF), which normally mediates angiogenesis; as a result, diminuted capillary density occurs, which along with the dysfunctional energy metabolism of cardiomyocytes progress to the development of cardiomyopathy [83,84]. Moreover, high expression of von Willebrand factor (vWF), which is a biomarker of endothelial damage, results in worse endothelial function reflected by deteriorated flow-mediated dilatation (FMD) [85,86].

### 3.2. Molecular Mechanisms of Endothelial Dysfunction in Patients with Heart Failure

#### 3.2.1. Heart Failure with Reduced Ejection Fraction

Coronary microvascular ED has been linked to asymptomatic LV dysfunction and could be an early step in the pathophysiology of systolic HF [87]. On the other hand, peripheral ED is a risk factor for the development of stage B HF, which is defined as the asymptomatic stage of HF with impaired systolic function [88]. Interestingly, even low-risk individuals of both sexes but with ED have a greater risk for the development of stage B HF, although the exact pathophysiologic mechanisms of cardiac remodeling have not been determined [88]. Possibly, the diminuted NO production could result in LV systolic dysfunction and the progression of HF syndrome [88]. The association between endothelial function and HF is thought to be more complex than the already known NO-mediated effects in the cardiovascular system; as a matter of fact, ED is a key characteristic of several circulatory beds in HF, irrespectively of LVEF [89]. Moreover, biomarkers of endothelial glycocalyx degradation, such as elevated heparin sulfate, have also been linked to HFrEF and worse prognosis [90,91]. What is more, ED occurs at a late stage in patients with HFrEF in contrast to HFpEF [4]. Lastly, according to the Multi-Ethnic Study of Atherosclerosis, impaired FMD of the brachial artery has been related to the risk of developing HF, and especially HFrEF, independently of the classic risk factors and natriuretic peptides levels [92].

Interestingly, polymorphisms of eNOS might be implicated in the risk of systolic HF in certain populations, whereas ethnic differences between microvascular and macrovascular ED have also been recorded [93,94,95]. Moreover, depletion of inducible nitric oxide synthase (iNOS) in an experimental study of wild-type mice with chronic transverse aortic constriction beneficially affect cardiac hypertrophy, dilation, fibrosis, and dysfunction, implying the detrimental effects of iNOS dysregulation in the maladaptive response to systolic overload of the heart [96].

On the other hand, oxidative stress plays an important role in the pathophysiology of ED in both systolic and diastolic HF; while low concentrations of ROS are normal during cellular function, ROS overexpression damages cellular gene expression and signaling pathways, which affects cardiac mechanics and energy utilization [97]. Additionally, ROS overproduction is implicated in the development of myocardial hypertrophy and dilation [98]. HFrEF is characterized by dysfunction of the NO-sCG-cGMP pathway, which implies the presence of ED; oxidative stress induces deleterious effects in the enzymes of this system, which end in deteriorated NO production and development of myocardial injury according to preclinical and clinical data [99,100]. As a matter of fact, treatment with agents with established antioxidant properties, such as allopurinol, has displayed beneficial effects in markers of systolic dysfunction of patients with HF, such as global longitudinal peak strain [98]. Moreover, activation of the nuclear factor kappa B (NF-κB) pathway and release of pro-inflammatory molecules such as intracellular adhesion molecule-1 (ICAM-1) and vascular adhesion molecule-1 (VCAM-1) is evident in systolic HF according to studies [101,102]. Additionally, other data indicate that decreased adiponectin levels are related to diminuted NO production in patients with systolic HF and the severity of HF [103].

Patients with chronic systolic HF display not only ED but also abnormal ventricular-arterial uncoupling, which is the pressure–volume interaction between LV and the vascular system [104]. Ventricular–arterial uncoupling has been associated with dysfunction of the mechanisms of cardiac energetics, pump efficiency, and poorer clinical outcomes [105]. Lastly, mineralocorticoid receptors of the endothelial cells have been related to the transition of cardiac hypertrophy to systolic dysfunction of the heart independently of pressure-induced overload damage and LV remodeling [106].

Finally, patients with systolic HF and pulmonary arterial hypertension display low numbers of EPCs and increased levels of osteoprotegerin, implying the deleterious effects of osteoprotegerin in the development of pulmonary ED and worsening the prognosis of systolic HF [107]. Similarly, according to another study, high levels of osteoprotegerin, diminuted EPCs, and increased mean pulmonary artery pressure have been associated with the damage induced by hypoxemia in patients with sleep-disordered breathing; as a result, it is speculated that ED and vascular remodeling of pulmonary vasculature orchestrate the deterioration of systolic function in HF [108].

#### 3.2.2. Heart Failure with Preserved Ejection Fraction

ED induces LV diastolic dysfunction, which is a key characteristic of both HF (and, in particular, HFpEF) and coronary artery disease [109,110]. Decreased microvascular reactivity in patients with diastolic HF implies that microvascular ED is responsible for the progression of subclinical heart remodeling [111]. Furthermore, ED is an important contributor to the development of LV diastolic and right ventricular dysfunction in patients with end-stage renal disease, whose volume status is normal [112]. Interestingly, a study in mice revealed that the induction of endothelial permeability was associated with the provocation of diastolic dysfunction and deterioration of cardiac function due to the disruption of endothelial cell–cardiomyocyte interactions and decreased ECM protein synthesis [113]. Moreover, overexpression of the human β3 adrenergic receptor in a transgenic rat model was linked to diminuted NOS3 mRNA expression, diastolic dysfunction of the aging heart, and reduced aortic flow upon diastolic stress [114]. Additionally, deterioration of the NO-cGMP pathway is implicated in the pathophysiology of diastolic HF since a dysfunctional endothelium is related to repeated episodes of ischemia/reperfusion and the development of a chronically stunned myocardium with systolic dysfunction and increased diastolic stiffness [68]. Nonetheless, a study in knockout mice for nuclear factor (erythroid-derived 2)-like 2 demonstrated that the development of LV diastolic dysfunction is attributed to SERCA2 a downregulation and not to the changes in coronary vascular function or systemic hemodynamics, which were preserved by compensatory upregulation of eNOS expression in the aorta and the heart [115].

Another important pathophysiologic aspect to be mentioned is the impact of diabetic metabolic derangement on LV function; interestingly, according to an experimental study, the effects of metabolic syndrome (hyperglycemia, hypercholesterolemia, and hypertriglyceridemia) were related to eNOS uncoupling, excessive nitroso-redox balance, alteration in genes of glucose and fatty acid metabolism as well as mitochondrial dysfunction [116]. Similarly, in another study of prediabetes, poor coronary endothelial function was linked to increased protein kinase C activity, mitochondrial oxidative stress, as well as rho-kinase-impairment of myosin head extension to actin filaments [117]. Additionally, a decrease in soluble guanylyl cyclase/PKG activity and stiffness of myocardial titin was evident, too [117]. Interestingly, epicardial adipose tissue demonstrates a positive association with cardiac structural and protein alterations, ED, reduced insulin sensitivity, and inflammation; possibly, local mechanic or paracrinic effects of epicardial fat could justify these results [118]. Furthermore, impaired expression of endothelial sirtuin-6 in diabetes mellitus has been linked to the dysregulated fatty acid transportation across the endothelium, which might contribute to the pathophysiology of HFpEF [51]. Finally, according to recent data, miR-30 d/e has been associated with the presence of diastolic dysfunction, impaired free fatty acid metabolism, and microvascular dysfunction in diabetes [119].

As for the role of inflammation, it should be mentioned that several risk factors induce a chronic, pro-inflammatory environment, which is responsible for the stimulation of the immune response, the perpetuation of hypoxemia, and the activation of neurohormonal systems; as a result, coronary microvascular ED develops and, subsequently, diastolic dysfunction of the heart [112,120]. As a matter of fact, circulating pro-inflammatory biomarkers such as IL-6 and CRP have displayed an association with echocardiographic parameters of diastolic dysfunction in HF [121]. Interestingly, according to a recent study in female mice, a deficiency of endothelial sirtuin-3 is responsible for the induction of diastolic dysfunction in the aged heart as well as for the elevation of blood pressure [122].

Evidence from basic science and clinical studies has revealed that systemic and myocardial oxidative stress derived by the enzyme nicotinamide adenine dinucleotide phosphate (NADPH) oxidase is an important contributor to LV diastolic dysfunction [123]. Moreover, according to an experimental study of pulmonary hypertension, diastolic HF has been associated with the lack of normal endothelial function as well as the development of increased pulmonary vascular resistance, vascular thickness, and biventricular cardiac hypertrophy [124]. Finally, according to other data, angiotensin II has been linked to the dysfunctional mechanisms of angiogenesis through effects in the Akt pathway, which ultimately result in the development of diastolic HF [125].

#### 3.2.3. Heart Failure of Ischemic Etiology

Acute myocardial ischemia (i.e., post MI) may result in scar formation, triggering cardiac remodeling and the development of HF [126]. Ischemic and non-ischemic HF display differences regarding their relation to ED [127]. Specifically, patients with ischemic HF usually have systemic ED, which involves arteries and veins, microcirculation, as well as coronary, pulmonary, and peripheral vessels [128]. Interestingly, according to a study, peripheral endothelium-dependent and endothelium-independent function was more deteriorated in patients with ischemic HF than in patients with non-ischemic HF [129]. Furthermore, a study by our research team has revealed that patients with ischemic HF have impaired FMD, and there is a linear improvement of FMD according to LVEF, while impaired endothelial function was associated with a worse cardiovascular prognosis [130].

Regarding the pathophysiology, according to a protein network analysis from patients with ischemic and non-ischemic HF, upregulation of 18 proteins related to the pathways of inflammation, ED due to superoxide production, coagulation, and atherosclerosis was evident in ischemic HF [126]. Surprisingly, five key network proteins such as acid phosphatase 5, epidermal growth factor receptor, insulin-like growth factor binding protein-1, plasminogen activator urokinase receptor, and secreted phosphoprotein-1 could discriminate ischemic HF from non-ischemic HF [126]. The involvement of inflammation in the development of post-MI HF has already been established; according to an experimental study in mice, angiotensin II exerts myocardial damage post-MI through the attachment of the pro-inflammatory Nox2+ myelomonocytic cells, macrophages, and monocytes at the vessel wall along with stimulation of oxidative stress and the ED [131]. Other important pro-inflammatory mediators in the pathophysiology of ischemic HF are CRP, pentraxin-3, osteoprotegerin, BNP, neopterin, and soluble suppression of tumorigenesis-2 (sST2) [132]. As a matter of fact, sST2, which is a biomarker of fibrosis, has exhibited higher expression in patients with ischemic HF and has been linked to the functional capacity of the patients; moreover, this biomarker is inversely associated with FMD of the brachial artery [133]. Finally, according to another study, endothelial function in patients with ischemic HF is further impaired compared to patients with dilated cardiomyopathy, implying the involvement of underlying atherosclerosis in the pathophysiology; indeed, patients with ischemic HF displayed higher levels of IL-6 and TNF-α [134].

As for oxidative stress, increased ADMA levels, which antagonize NO production and are a key characteristic of ED, were associated with poor cardiovascular prognosis in patients with ischemic HF [135]. Moreover, increased thrombogenicity, ED, and oxidative stress have been implicated in the development of atrial fibrillation in ischemic HF; specifically, evidence from a study in mice exhibited decreased expression of atrial eNOS, SERCAa, thrombomodulin, tissue factor pathway inhibitor, and tissue plasminogen activator [135]. Additionally, at the molecular level, exosomes (which are vectors for intracellular communication) are associated with ischemic heart disease and its evolution to HF through the mechanisms of ED, lipid accumulation, atherosclerotic plaque development, and ischemia–reperfusion injury [136,137].

Post-MI remodeling is a detrimental consequence of MI; interestingly, in a study of patients with ischemic HF, increased mRNA levels of adrenomedullin exhibited a relation with post-ischemic myocardial remodeling [138]. Similarly, dysfunction of the T-regulatory cells has also been implicated in the development of chronic ischemic HF through immune system activation and LV remodeling [139]. What is more, mineralocorticoid receptors and RAAS activation are important determinants of post-MI LV dysfunction; specifically, according to a study in mice, deletion of mineralocorticoid receptors in Vascular smooth muscle cells (VSMCs) could ameliorate LV dysfunction post-MI through control of coronary flow reserve and improvement of endothelial function [140].

As for EPCs, increased circulating levels of EPCs along with FMD could predict LV remodeling post-MI and the occurrence of major adverse cardiovascular events [141]. Interestingly, insulin resistance in diabetic patients with ischemic HF has been linked to decreased circulating numbers of proangiogenic EPCs [141]. Additionally, the reduced amount of CD14(+)CD309(+)-and CD14(+)CD309(+)Tie2(+) circulating EPC was related to the severity of LV dysfunction in patients with ischemic HF, whereas CD45(+) CD34(+) and CD45(-) CD34(+) mononuclear cell counts were associated with the severity of coronary artery lesion [142]. Last but not least, increased endothelial-derived apoptotic microparticles in patients with ischemic HF are associated with ED and poor prognosis [143].

#### 3.2.4. Non-Ischemic Heart Failure

Non-ischemic HF is a primary disease of cardiomyocytes and interstitial space [144]. The use of multiple circulating biomarkers might be used to reveal possible pathophysiologic pathways that are linked to a certain phenotype of HF (ischemic vs. non-ischemic) [126]. As for ED, there is evidence that peripheral endothelial function is not impaired in patients with non-ischemic HF [145]. Indeed, the pattern of ED in patients with non-ischemic HF is more heterogenous and exhibits fewer systemic abnormalities, whereas ED of coronary circulation occurs more frequently [128]. Abnormal coronary microvascular flow has been associated with deteriorated myocardial perfusion and consequent metabolic changes in cardiomyocytes, which trigger local myocardial ischemia [146,147]. Other studies have also confirmed the heterogenous nature of microvascular ED in patients with LV dysfunction of unknown cause [146]. Coronary endothelial-independent microvascular dysfunction has been related to higher brain natriuretic peptide (BNP) levels and ventricular wall tension in patients with non-ischemic HF, especially in those with cardiac fibrosis [148].

As for the pathophysiology, diabetes mellitus is associated with the development of diabetic cardiomyopathy irrespectively of the ischemic damage through complex effects on vascular endothelial function; specifically, hyperglycemia increased free fatty oxidation, decreased NO production, oxidative stress, inflammation, and dysfunctional endothelial permeability contribute to the pathophysiology of diabetic cardiomyopathy [149]. Additionally, according to the literature, patients with non-ischemic HF display increased myocardial expression of vWF (which is a glycoprotein produced by endothelial cells that control platelet aggregation and thrombus formation at the sites of vascular injury), implying the impact of ED-derived vWF release in the development of non-ischemic HF [150].

On the other hand, in patients with non-ischemic HF, NO and a secondary endothelium-derived relaxing factor sensitive to high K+ have demonstrated vasodilative properties [151]. In general, ED impairs LV systolic function due to the increase in systemic vascular resistance; then, LV dysfunction further deteriorates endothelial function through decrease in shear stress and NO bioavailability [152]. Interestingly, in a study of patients with systolic, non-ischemic HF, LVEF was related to ED, implying the importance of the management of ED in these patients [152]. Lastly, NO inhibition of the peripheral vasculature in patients with non-ischemic HF resulted in higher basal vascular tone and the progression of the disease [153].

Therefore, the involvement of ED in the pathophysiology of non-ischemic HF is not consistent among the studies suggesting that further research should be performed in order to elucidate the exact impact of ED on non-ischemic HF.

#### 3.2.5. Right Heart Failure Complicating Pulmonary Arterial Hypertension

In pulmonary arterial hypertension (PAH), the increase in pulmonary vascular resistance is responsible for the rise in right ventricular afterload and the progression to right HF [154]. The abnormal hypertrophy of small pulmonary arteries ranges from hypertrophy and hyperplasia of the media layer to the excessive apoptosis and proliferation of pulmonary arterial smooth muscle cells, which end in the formation of plexogenic lesions that obstruct artery lumen and decrease pulmonary blood flow [155]. Importantly the progressive occlusive arterial remodeling of pulmonary arterioles is characterized by the presence of significant ED [156,157].

Interestingly, it has been proposed that ED of pulmonary circulation displays an association with ED of the systemic circulation, implying that PAH is a situation of global vasculopathy [158]. Pulmonary artery endothelial cells are vulnerable to several insults (such as toxins, hypoxia, pro-inflammatory cytokines, and shear stress), which, along with the presence of genetic susceptibility, are responsible for the pathophysiology of disease; increased shear stress, in particular, develops an environment of raised arterial pressure and fluid dynamics which end in endothelial cell injury [159,160]. As a result of pulmonary artery endothelial cell damage, excessive oxidative stress, hyperproliferation, and coagulation occur [159]. Moreover, at the later stages of endothelial cell damage, apoptosis-resistant endothelial cells develop along with excessive angiogenesis [161,162]. Interestingly, research has revealed that pulmonary microvascular endothelial cells have intrinsic deficits that hinder proper response to Vascular endothelial growth factor A stimulation [155]. Another pathophysiologic characteristic is the poor tolerance of pulmonary microvascular endothelial cells to hypoxic injury [155]. Moreover, evidence from experiments in mice has revealed that inhibition of Hypoxia-inducible factor 1α could decrease right ventricular systolic pressure and hypertrophy as well as the amount of fibrosis and obliterative pulmonary vascular remodeling [163].

Endothelial cell permeability is compromised in PAH and, under the expression of growth factors such as VEGF, activation of pro-inflammatory mediators and cytokines occurs [164]. As a result, inflammation perpetuates vascular damage and endothelial barrier permeability, which is responsible for the distorted gas exchange and coagulation between lung and blood tissue [165]. Moreover, crosstalk between endothelial cells and VSMCs, as well as with non-smooth muscle cells, results in the chemoattraction of immune cells at the sites of vascular damage such as myofibroblasts and pro-inflammatory leucocytes [166]. Another important pathophysiologic aspect is endothelial-to-mesenchymal transition in which endothelial cells transform into a profile that resembles myofibroblast or mesenchymal cells; as a matter of fact, endothelial cells lose the expression of their typical markers such as CD31 and cadherins and exhibit proliferation of α-smooth muscle actin and vimentin [167]. According to the studies, the transforming growth factor-β (TGF-β) signaling pathway mediates the expression of Smooth muscle alpha-actin (αSMA) and type I collagen (and not VE-cadherin) in pulmonary arterial endothelial cells [168,169]. Endothelial-to-mesenchymal transition is controlled epigenetically by several miR such as miR-21; for instance, TGF-β augments miR-21 expression in endothelial cells through AKT-dependent pathway [170]. Also, there is evidence that miR affect ion expression, mitochondrial function and are implicated in the angiogenic impairment in PAH [171]. 

A genetic involvement is present in 6–10% of patients with PAH and most commonly involves the heterozygous germline mutation of the *Bone Morphogenetic Protein Receptor* (*BMPR)* gene, which encodes type 2 bone morphogenetic protein receptor (BMPR-2) [172]. There is evidence that autophagy in the lysosomes of human pulmonary artery endothelial cells might contribute to BMPR-2 deletion in PAH [173]. Additionally, control of gene expression at the transcriptional, post-translational, and post-transcriptional level by long non-coding RNAs is implicated according to recent studies in the development of pulmonary vascular remodeling through effects in endothelial function, cell proliferation, angiogenesis, endothelial-to-mesenchymal transition and cellular metabolism [174]. Last but not least, improper NO release due to ED induces DNA damage and metabolic dysregulation [175]. Indeed, it has been described that pulmonary artery endothelial cells and adventitial fibroblasts display a shift from glucose oxidation towards uncoupled aerobic glycolysis, which hinders the contractility of cardiomyocytes [176]. Moreover, metabolic abnormalities in PAH include irregular polyamine and sphingosine metabolism, impaired insulin sensitivity, and poor iron handling [177]. Gene expression studies indicate that human pulmonary artery smooth muscle cells exhibit increased fatty acid metabolism, formation of unsaturated fatty acids as well as a phenotype of the energy-driven proliferative profile along with decreased expression of the genes of the tricarboxylic acid cycle [178,179]. For instance, the deletion of peroxisome proliferator-activated receptor γ (PPARγ) results in systolic dysfunction of both ventricles and lipid accumulation inside cardiomyocytes [180]. Finally, a disintegrin and metalloproteinase with thrombospondin motifs 8 (ADAMTS8) (disintegrin and metalloproteinase with thrombospondin motifs 8) is implicated, according to experimental evidence, in mitochondrial fragmentation under hypoxia as well as with the proliferation of pulmonary artery smooth muscle cells [181].

### 3.3. Endothelial Function in the Course of Heart Failure

Impaired endothelial function in patients with HF is associated with poorer cardiovascular outcomes, implying proper therapeutic management of ED [182]. ED is more severe in HFpEF and appears earlier during the progression of HFpEF; contrarily, ED is evident at a later stage in HFrEF [4]. Moreover, ED of the peripheral vasculature is maximal during the early stages of HF in contrast to more severe stages of this syndrome [183]. Patients with acute HF are characterized by the presence of a procoagulant state due to ED, which is linked to poorer cardiovascular prognosis [184]. Deteriorated peripheral endothelial function may also predict long-term cardiovascular events in patients with end-stage HF and could facilitate risk stratification strategies in these patients [185]. Similarly, impaired peripheral endothelial function and decreased exhaled NO (as a marker of pulmonary circulation) during submaximal exercise in patients with chronic HF have been linked with higher mortality after adjustments for clinical factors [186]. Finally, according to another study, evaluation of ED in patients who receive treatment with cardiac resynchronization therapy (CRT) might also indicate the patients with better response [187].

On the other hand, there is evidence that not only peripheral but also coronary microvascular and epicardial dysfunction are associated with the clinical outcomes in HF; as a matter of fact, preservation of endothelial function might improve LVEF in patients with HF [188]. Coronary microvascular ED drives the development of HFpEF due to the existence of an environment of chronic, subclinical inflammation [188]. Interestingly, circulating inflammatory markers have been related to coronary microvascular ED (assessed by transthoracic Doppler echocardiography), as well as with markers of diastolic dysfunction, such as the increased E/e’ ratio [188]. Similarly, in a study of patients with HFpEF, oxidative stress reflected by the levels of increased MPO, uric acid, calprotectin, and symmetric dimethyl arginine, are associated with diastolic dysfunction; as a matter of fact, microvascular ED was linked to worse cardiovascular prognosis [189].

**Figure 1:**
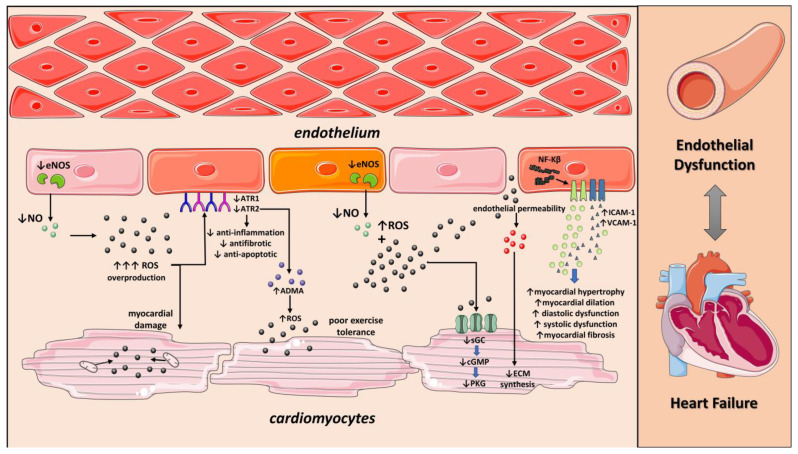
Role of Endothelial dysfunction on Heart failure. Endothelial dysfunction induces impaired NO production and ROS overproduction. Excessive ROS increases ADMA levels resulting in decreased exercise tolerance. Moreover, ROS production at mitochondria and the presence of dysfunctional antioxidant system cause hypoxia and accelerate myocardial damage. Deterioration of NO-cGMP-PKG pathway increases diastolic dysfunction, myocardial fibrosis, and myocardial hypertrophy. NO: nitric oxide; ROS: reactive oxygen species; eNOS: Endothelial nitric oxide synthase; ADMA: Asymmetric Dimethylarginine; ATR1: Angiotensin II receptor type 1; ATR2: Angiotensin II receptor type 2; NF-Kβ: Nuclear factor kappa beta; ICAM-1: Intercellular Adhesion Molecule 1; VCAM-1: vascular cell adhesion molecule 1; cGMP: Cyclic guanosine monophosphate; sGC: soluble guanylate cyclase; PKG: protein kinase G. Parts of the figure were drawn by using pictures from Servier Medical Art. Servier Medical Art by Servier is licensed under a Creative Commons Attribution 3.0 Unported License (https://creativecommons.org/licenses/by/3.0/ accessed on 23 of December 2022).

Patients with acute and chronic HF exhibit various degrees of ED and circulating biomarkers of endothelial activation [190]. Interestingly, a study in hypertensive patients revealed that impaired endothelial function and high CRP levels are associated with the development of new-onset HF [191]. Furthermore, according to recent data, high circulating levels of biomarkers of endothelial glycocalyx impairment are linked to increased mortality in patients with decompensated HFrHF, implying the role of dysfunctional endothelium for poor prognosis [90]. Moreover, ED could predict the incidence of adverse events in acute HF as well as HF progression [68]. Moreover, the identification of ED might reveal individuals at risk for developing HF and facilitate the therapeutic monitoring of those who already receive cardiotoxic agents [88]. Interestingly, according to another study by our research team, FMD could serve as a risk-stratification tool in order to evaluate anthracycline-induced cardiotoxicity in patients who receive chemotherapy [192] (Figure 1).

## 4. Modification of Endothelial Dysfunction in Patients with Heart Failure

### 4.1. Effects of Exercise Training

Exercise and cardiac rehabilitation have demonstrated beneficial effects in patients with HF and especially in HFrEF; as a matter of fact, HFrEF is characterized by exercise intolerance, and several assessments could evaluate exercise capacity in these patients, such as a 6-minute walk test and peak oxygen uptake (VO2) [193]. It should be pointed out, though, that not all patients are capable of cardiac rehabilitation, and those who are appropriate candidates achieve an improved quality of life, better exercise capacity, and fewer cardiovascular events [193]. The benefits of structured exercise training (ET) have been well established for HF and have been given a class IA recommendation for patients with stable HF [194,195]. Three modalities of ET have been proposed: endurance-aerobic (continuous or interval training, with superior effects of endurance on LV function), strength/resistance training (individually tailored to each patient’s needs), and respiratory training (preferred in circumstances of inspiratory muscle weakness) [196].

According to the literature, ET improves FMD through effects on shear stress as well as arterial compliance [197]. Specifically, evidence from a meta-analysis of 16 studies indicated that ET enhances NO bioavailability by assisting eNOS function and antioxidant enzymes expression, mobilization of EPCs, and decrease in TNF-α, IL-10, and IL-6 expression [197]. Interestingly, in the Leipzig Exercise Intervention in Chronic heart failure and Aging (LEICA) study, 4-week ET in patients with stable congestive HF improved FMD regardless of age; a rise in EPCs number and function was also documented [198]. Moreover, maximal cardiopulmonary exercise testing (CPET) in patients with chronic HF of different severity has displayed enhanced mobilization and circulation of EPCs, although the effect was irrelevant to the disease severity [60]. ET, according to another study, demonstrated favorable effects in LVEF of elderly patients with chronic HF, which was mediated by mobilization of EPCs, stimulation of NO and VEGF expression, as well as of PI3 K/AKT pathway of angiogenesis [199]. Interestingly, 12 weeks of high-intensity interval exercise (HIIT) in patients with HFrEF resulted in decreased SNS activity and better peripheral vascular function reflected by brachial artery FMD in contrast to moderate-intensity continuous training [200].

As for HFpEF, ET has indicated favorable effects and is considered to be an important non-pharmaceutic option, which improves the quality of life and exercise capacity through mechanisms that involve endothelial function, such as the regulation of inflammation [201]. In elderly patients with HFpEF, 16 weeks of ET had a beneficial effect on VO2 and quality of life without effects on FMD or arterial stiffness, and the possible mechanism could be better skeletal muscle perfusion or oxygen utilization [202]. According to a systematic review of 9 studies of patients with HFpEF, ET resulted in higher VO2 uptake, 6-minute walking distance, and improved ventilation threshold, although there was no significant effect on endothelial function and arterial stiffness; interestingly, only in some of the studies echocardiographic parameters and quality of life displayed improvement [195].

In conclusion, ET exerts beneficial effects in the appropriate candidates with HF, and the possible mechanisms involve the improvement of endothelial function and SNS activity, which result in enhanced exercise tolerance, possibly due to better diffusion and utilization of oxygen in skeletal muscles and oxygen transport to tissues [193].

### 4.2. Drugs for the Treatment of Endothelial Dysfunction in Patients with Heart Failure

#### 4.2.1. Statins

Statins, known as 3-hydroxy-3-methylglutaryl coenzyme A (HMG-CoA) reductases, display not only lipid-lowering actions against LDL levels but also pleiotropic actions such as anti-inflammatory, anti-atherosclerotic, and antioxidant actions [203]. In fact, these cardiovascular effects are independent of lipid-lowering actions and involve the mobilization of EPCs in circulation and enhancement of endothelial function [203]. According to the literature, statins improve LV remodeling and diastolic dysfunction of the heart as well as natriuretic peptide expression, possibly through the control of inflammation and restoration of endothelial function [204].

Patients with HFrEF statins displayed increased mobilization of EPCs, which further improved the exercise capacity and morbidity of the patients [205]. Additionally, statins decrease TNF-α levels and augment NO bioavailability through the stabilization of the mRNA of eNOs synthase [205]. Similarly, in another study, short-term administration of rosuvastatin, but not allopurinol, increased the number of circulating EPCs in patients with systolic HF, although EPCs did not display associations with biomarkers of oxidative stress and inflammation [206]. Furthermore, in a study of patients with congestive HF administration of statins activated circulating CD34+ EPCs and enhanced the neovascularization process, which was reflected by higher expression of VEGF, improved endothelial function, and LVEF [207]. In patients with congestive HF, atorvastatin restored peripheral endothelial function assessed by gauge-strain plethysmography and decreased the expression of TNF-α, IL-6, and VCAM-1 [208]. Higher doses of atorvastatin, in contrast to the lower doses, induced favorable effects on endothelial function in patients with ischemic HF and improved FMD, augmentation index, as well as MMP-9 and ICAM-1 levels [209]. Last but not least, the administration of rosuvastatin in patients with congestive HF raised FMD in contrast to the administration of ezetimibe, although there were no differences in lipid levels between study arms [210].

#### 4.2.2. Beta Blockers

Beta-blockers (β blockers) belong to the antagonists of beta-adrenergic receptors, which are normally expressed in cardiomyocytes and mediate the actions of SNS [211]. Beta-blockers bind to β1, β2, and β3 receptors of the G-protein-coupled receptors family; the first generation of β blockers is non-selective for β1 receptors, the second-generation of beta blockers is more cardio-selective, and the third generation is highly selective for β1 receptors [211]. As a matter of fact, the third generation of β blockers exhibits vasodilatory properties along with antioxidant, anti-proliferative, anti-hypertrophic, angiogenic, and anti-apoptotic properties, which are under investigation [211]. Therefore, β blockers with NO-mediated vasodilatory properties could be a promising treatment for the restoration of ED [212]. Moreover, according to clinical trials, β blockers display a survival benefit for patients with chronic HF, too [213].

Interestingly, in the study of Chin BSP et al., a three-month scheme of β blockers in patients with chronic HF resulted in marked improvement of biomarkers of lipid peroxidation without changes in total antioxidant capacity and vWF levels [214]. In another study in patients with congestive HF and NYHA II-III, administration of bisoprolol for 20 ± 10 weeks could also restore ED [215]. Similarly, a 3-week regimen of carvedilol in patients with HF and NYHA II-III improved L-arginine and L-citrulline levels and decreased the expression of VCAM-1, implying a beneficial effect in endothelium-dependent dilatation, fibrinolysis and hemorheological profile of patients [216]. Furthermore, Poelzl G. et al. displayed that short-term administration of β blockers and ACE inhibitors in patients with chronic HF improved FMD and submaximal exercise capacity [217]. Additionally, treatment with carvedilol for six months could improve HF functional class, LVEF, 6-minute walk distance in patients with chronic HF (without effects in peak VO2 max), and plasma malondialdehyde levels [218]. What is more, in a study of patients with HF and NYHA II-IV, administration of carvedilol for 40 ± 14 months ameliorated HF symptoms and the expression of pro-inflammatory biomarkers irrespectively of LVEF; interestingly, patients who exhibited an improvement of LVEF had also decreased ADMA levels [219]. As for the effects of specific β blockers, the switch from carvedilol to either metoprolol tartrate or succinate in patients with mild HF did not affect endothelium-dependent vasodilation, blood pressure, or heart rate [220]. Last but not least, combination of sacubitril/valsartan and metoprolol in patients with congestive HF ameliorated LV end-systolic and end-diastolic dimensions, LVEF, biomarkers of oxidative stress and coagulation parameters, implying possible effects in cardiac remodeling [221]. In conclusion, the majority of studies underline that β blockers might restore ED in HF, and to our knowledge, only one study in patients with chronic HF has demonstrated neutral effects of β blockers on markers of endothelial, platelet, or hemorheological function [222].

#### 4.2.3. ACE Inhibitors/ARBs

Angiotensin-converting enzyme inhibitors (ACEi) are the mainstay drug category of patients with HF due to their antihypertensive and anti-atherosclerotic properties, including the ability to delay the progression of LV remodeling [223]. Concerning their effect on endothelial function, chronic administration of ACEi in patients with congestive HF has proven beneficial through the improvement of FMD, compliance, and distensibility of the brachial artery, possibly due to their blood-pressure-lowering actions [224]. According to another study, administration of ACEi but not beta-blockers in patients with chronic HF resulted in a more controlled hypercoagulable state, which was reflected by the decreased levels of soluble P-selectin, vWF, and fibrinogen, especially in females and in patients with more progressed NYHA status [222]. Interestingly, treatment of patients with chronic HF with ramipril and sildenafil (both solely and in combination) improved FMD, and this effect remained significant at 4-hour post intervention [225]. Lastly, a recent study that investigated patients with HFmrEF and HFpEF demonstrated that therapy with perindopril for 12 months ameliorated endothelial function of large blood vessels and microvessels (assessed by the method of photoplethysmography); interestingly, both categories of HF exhibited decreased expression of E-selectin, whereas ET-1 had the maximal improvement in patients with HFpEF [226].

Angiotensin Receptor Inhibitors (ARBIs) selectively block the AT-1 receptor pathway, which mediates their antihypertensive functions; as a result, angiotensin-II binds to the AT-2 receptor, which possesses atheroprotective functions [227]. ARBIs do not affect the bradykinin pathway, which is characteristic of ACEi effects on endothelial continuity [227]. Angiotensin-II induces deleterious effects on endothelial function, which involve the senescence of EPCs due to oxidative stress and telomerase inactivation [228]. ARBIs, in general, have beneficial actions against several atherosclerotic diseases, including HF [33]. Nevertheless, their exact effects on endothelial function are less well understood [227]. For instance, according to a study of patients with congestive HF, ACEi or AT-II antagonists improved flow-dependent vasodilatation, shear stress, as well as compliance and distensibility of the radial artery [229].

#### 4.2.4. Mineralocorticoid Receptor Antagonists (MRA)

Aldosterone is a mineralocorticoid hormone with detrimental effects on endothelial cells and cardiomyocytes; specifically, aldosterone is implicated in cardiac hypertrophy and fibrosis in HF in addition to direct vascular injury [230]. Aldosterone also hinders the function, growth, and mobilization of EPCs in a concentration-dependent manner through VEGF-mediated phosphorylation of the Akt pathway [230,231]. MRAs, such as eplerenone and spironolactone, neutralize the harmful effects of aldosterone on the cardiovascular system [231]. Indeed, treatment with spironolactone restores endothelial function through augmentation of NO bioavailability and endothelium-dependent vasodilation in patients with NYHA class II-III chronic HF under standard diuretic/ACEI therapy [232]. Similarly, in another study, the administration of spironolactone in patients with congestive HF improves FMD at 4 weeks, and these effects remain at 8 weeks, possibly due to the attenuation of aldosterone actions in endothelial function [233]. Finally, in a recent study, Levi et al. displayed that 8-week treatment with eplerenone or spironolactone in patients with congestive HF raised VEGFR2+/CD34+ and VEGFR-2+/CD133+ levels of circulating EPCs, implying the beneficial effects of MRA antagonism for maintenance of endothelial function [230].

#### 4.2.5. SGLT2 Inhibitors

The novel drug category of sodium-glucose cotransporter 2 (SGLT2) inhibitors has cardioprotective effects in patients with HF through various mechanisms, including improvement of endothelial function, aside from their established effectiveness in the treatment of diabetes mellitus [234]. SGLT2 inhibitors act on the renal proximal tubule, reduce glucose reuptake, and promote sodium excretion leading to glycosuria, natriuresis, and diuresis [235]. According to the literature, the intracellular decrease in sodium levels is responsible for the cardioprotective actions of SGLT2 inhibitors in HF through amelioration of calcium ion handling in cardiomyocytes; as a result, there is an enhancement of the electromechanical function of the heart [235]. Other mechanisms involve control of oxidative stress, fibrosis, autophagy, and inflammation [235]. Evidence from experimental studies indicates that SGLT2 inhibitors counteract mitochondrial dysfunction (due to energy starvation in HF), activate the sirtuin-1 pathway and stimulate ketogenesis [236,237]. Apart from the direct effects of SGLT2 inhibitors in cardiomyocytes, many of their actions involve the regulation of ED, diastolic dysfunction, cardiac stiffness, and reduction of epicardial tissue [238,239].

Empagliflozin improves eNOS-dependent PKGIα oxidation and decreases the expression of ICAM-1, VCAM-1, IL-6, and TNF-α in myocardial tissues of patients with HFpEF [240]. Additionally, empagliflozin enhances the phosphorylation of myofilament proteins and the NO-cGMP pathway, which reflects its antioxidant effects [240]. Cardiomyocytes and macrophages, when treated with empagliflozin, present activation of the AMK kinase pathway as well as inhibition of iNOS function [241]. Data from clinical studies are yet scarce; nevertheless, according to an observational, nonrandomized study, the administration of empagliflozin in diabetic patients with chronic HF improves FMD [182].

#### 4.2.6. Sacubitril-Valsartan (ARNIs)

Sacubitril-valsartan, an angiotensin-receptor/neprilysin inhibitor (ARNI), is a drug category for chronic symptomatic HFrEF and HFpEF according to the recent guidelines for HF [242]. Sacubitril is a pro-drug, and its activated metabolite inhibits neprilysin from breaking down natriuretic peptides; as a result, vasodilation, natriuresis, and diuresis occur, which are associated with enhanced endothelial function [242]. Blockage of neprilysin increases bradykinin, which possesses endothelium-dependent vasodilatory actions, too [242]. On the other hand, valsartan is an ARB that blocks the RAAS system and protects against vasoconstriction, hypertension, and cardiac remodeling in HF [242,243]. Nevertheless, neprilysin disintegrates angiotensin II; therefore, sacubitril should be used along with an ARB in order to counteract high levels of angiotensin II [242,243].

Considering the effects of sacubitril-valsartan on endothelial function, Amore et al. indicated that treatment with sacubitril-valsartan for six months in patients with dilated cardiomyopathy and reduced LVEF restore endothelial function, LVEF, diastolic dysfunction, and mitral regurgitation, without any significant effects in arterial stiffness [244]. Interestingly, a study in 80 patients with HFrEF proves that sacubitril-valsartan added on standard-of-care regimens for a period of 12 weeks augment FMD; furthermore, an increase in NO and NOS levels, LVEF and calcitonin gene-related peptide was found as well as decreased expression of ET-1 [245]. Lastly, sacubitril-valsartan administration for 12 weeks on top of conventional treatment improved FMD as well as arterial stiffness of patients with HF, too [246] (Table 1).

#### 4.2.7. Endothelin Receptor Antagonists (ERAs)

ET-1 is a powerful vasoconstrictor involved in several cardiovascular diseases, including PAH and congestive HF, and participates in the processes of cardiac hypertrophy, inflammation, and atherosclerosis [259]. ET-1 exerts vasoconstrictive and pro-inflammatory actions upon binding to ETA receptors of smooth muscle cells and contradictory actions upon binding to ETB receptors of pulmonary artery endothelial cells, which trigger the clearance of ET-1 from circulation and the release of endogenous NO and prostacyclin [260,261]. Therefore, blockage of ETA receptors could be a promising therapeutic option against PAH, and currently three endothelin receptor antagonists (ERAs)—bosentan, ambrisentan, and macitentan—have been evaluated in clinical trials and have displayed favorable effects in PAH [259,262]. According to evidence from in vitro studies, bosentan improves endothelial function and decreases neointimal and smooth muscle cell proliferation in PAH [263]. Furthermore, evidence from in vivo studies in pigs have demonstrated that bosentan might partially improve hypoxia-related decrease in NO production [264]. Interestingly, administration of bosentan in humans with PAH for a period of six months resulted in ameliorated endothelial function of pulmonary microcirculation upon invasive assessment of endothelial function during right heart catheterization [263]. Similarly, in a study of patients with systemic sclerosis and no-PAH-related symptoms, bosentan reduced exercise-induced PAH; as a matter of fact, bosentan improved 6-minute walk indices, FMD of the brachial artery, and peripheral vasodilation [265]. Another study in patients with moderate to severe idiopathic PAH indicated that treatment with bosentan diminuted the expression of pro-inflammatory biomarkers such as ICAM-1, VCAM-1, IL-6 along with BNP and ameliorated the clinical status of the patients [256]. What is more, evidence from a study of patients with systemic sclerosis and PAH proved that a regimen of bosentan for 12 months could normalize the expression of ICAM-1, VCAM-1, P-selectin, and platelet/endothelial cell adhesion molecule (PECAM-1) and restore T-cell function [257]. Additionally, in patients with connective tissue diseases and PAH, a 3-month scheme of bosentan could ameliorate several biomarkers of endothelial function such as NO and sCD40 L and clinical status; interestingly, responders to the treatment demonstrated a decrease in P-selectin levels [266]. Last but not least, a study by Sfikakis PP et al. demonstrated that a 4-week scheme of bosentan in patients with systemic sclerosis could improve FMD of the brachial artery but not endothelium-independent vascular function; therefore, it has been speculated that this drug protects against systemic scleroderma-associated endothelial injury [258] (Table 2).

## 5. Conclusions

HF is a clinical syndrome with a diverse etiopathology but common pathophysiologic background. Several mechanisms are implicated in the course of HF, progression, and prognosis, with neurohormonal activation considered the key determinant of the syndrome. Endothelial dysfunction is a significant characteristic that accompanies HF of either etiology or type. Moreover, HF per se may worsen endothelial health through decrease in shear stress, stimulation of inflammation, inactivation of eNOS, and increased oxidative stress. Indeed, impaired endothelial function has been linked to deteriorated functional status and ventricular function in patients with HF. Importantly, most of the therapeutic options with established benefits in patients with HF have a parallel beneficial effect in the restoration of endothelial function.

## Figures and Tables

**Table 1:** Therapeutic options for the treatment of endothelial dysfunction in patients with heart failure.

Study	Study Design	Key Findings
***Exercise Training (ET)***		
Sandri M et al., 2016 [198]	RCT; 60 patients with chronic HF vs. 60 controls.	ET vs. control group improved FMD, CD34/KDR+ EPCs, and migratory capacity of cultured mononuclear cells.
Chen J et al., 2021 [199]	RCT; 80 patients with chronic HF and ΕΤ.	ET improved LVEF and LVFS. Additionally, ET had higher EPCs levels and proliferation ability and lower BNP levels and EPCs apoptosis rate.
Isaksen K et al., 2019 [132]	Controlled prospective trial; 30 patients with ischemic HF and ICD.	ET vs. control group improved peak VO2 and endothelial function.
Kitzman DW et al., 2013 [202]	RCT; 63 patients with HFpEF.	ET improved peak VO2 and quality of life but no endothelial function.
Angadi SS et al., 2015 [247]	RCT; 19 patients with HFpEF.	High-intensity interval training improved peak VO2, LV diastolic dysfunction. No effect on endothelial function was demonstrated.
***Statins***		
Oikonomou E et al., 2015 [205]	RCT; 26 patients with stable HF; evaluation of atorvastatin 10 mg/day vs. atorvastatin 40 mg/day for 4 weeks.	40 mg/day Atorvastatin demonstrated higher EPCs and FMD values and decreased TNF-α levels in both compared groups.
Tousoulis D et al., 2011 [206]	RCT; 60 patients with systolic HF; administration of rosuvastatin 10 mg/day vs. allopurinol 300 mg/day or placebo.	Rosuvastatin group had increased CD34/KDR+, CD34/CD133/KDR+ and EPCs levels. Additionally, improvement of endothelial was observed.
Erbs S et al., 2011 [207]	RCT; 42 patients with chronic HF randomized to 12 weeks of oral rosuvastatin (40 mg/d) or placebo.	Rosuvastatin 40 mg/day increased VEGF levels, CD34+ stem cell count, number of CD34/KDR+ EPCs, EPC integrative capacity, and FMD.
Tousoulis D et al., 2005 [208]	RCT; 38 patients with HF: Group 1: atorvastatin 10 mg/day (n = 19), group 2: control (n = 19), duration of treatment: 4 weeks.	Atorvastatin 10 mg/day improved forearm vasodilatory response to reactive hyperemia; decreased levels of IL-6, TNF-α, and sVCAM-1.
Tousoulis D et al., 2013 [209]	RCT; atorvastatin in 22 patients with ischemic HF.	Atorvastatin 40 mg/day vs. 10 mg/day significantly improved FMD and AIx values.
Winzer EB et al., 2016 [248]	RCT; 18 patients with chronic HF; randomized to 12 weeks of rosuvastatin vs. placebo	Rosuvastatin improved FMD and LDL cholesterol levels. Moreover, deterioration of FMD and LDL after cessation of therapy was detected.
***Angiotensin-converting enzyme inhibitors (ACEi)/Angiotensin Receptor Inhibitors (ARBs)***		
Gibbs CR et al., 2001 [222]	Cross-sectional study; 120 patients with chronic HF and sinus rhythm; n = 20 on lisinopril vs. n = 20 on β blocker (carvedilol or bisoprolol)	Initiation of beta-blocker therapy revealed no significant changes in hemorheological, endothelial function, and platelet indices. Moreover, initiation of ACEi decreased fibrinogen and vWF levels.
Hryniewicz K et al., 2005 [225]	RCT, 64 patients with chronic HF; randomization in placebo vs. 10 mg ramipril vs. 50 mg sildenafil vs. combination of ramipril/sildenafil.	Ramipril vs. placebo increased FMD at 4 h. Moreover, Sildenafil vs. placebo increased FMD at 1, 2, and 4 h. Combination of sildenafil/ramipril vs. placebo increased FMD at 1, 2, and 4 h.
Safonova JI et al., 2022 [226]	Cross-sectional study; 40 patients with HF (n = 20 patients with HFpEF; n = 20 patients with HFmrEF); administration of 12-months perindopril.	Perindopril increased in phase shift in both HFpEF and HFmrEF; increase in occlusion index in both HFpEF and HFmrEF; decreased E-selectin in both HFpEF and HFmrEF; ET-1 levels significantly decreased only in HFpEF
Ellis GR et al., 2002 [249]	RCT; 28 patients with HF on ACEi; randomization to candesartan vs. placebo	Candesartan vs. placebo has no effects on brachial artery FMD, exercise capacity (peak VO2), and biomarkers of oxidative stress.
Nakamura M et al., 2002 [250]	RCT; 26 patients with congestive HF that randomized to losartan vs. placebo.	Losartan group revealed increased forearm blood flow in response to intra-arterial infusion of acetylcholine.
***Mineralocorticoid Receptor Antagonists (MRAs)***		
Farquharson et al., 2000 [232]	RCT; 10 patients with chronic HF on standard diuretic/ACEi therapy were randomized to 50 mg/day spironolactone vs. placebo for 1 month.	Spironolactone improved forearm blood flow response to acetylcholine increased NO bioactivity, and inhibition of vascular angiotensin I/angiotensin II conversion.
Abiose AK et al., 2004 [233]	Cross-sectional; n = 20 patients with congestive HF; administration of spironolactone.	Administration of spironolactone improved FMD at 4 and 8 weeks.
Macdonald JE et al., 2004 [251]	RCT; 43 patients with congestive HF under ACEi and beta blockers; administration of 12.5-50 mg/day spironolactone vs. placebo for 3 months.	Administration of spironolactone increased acetylcholine-mediated vasodilatation and vascular ACE activity. Moreover, spironolactone decreased BNP and procollagen III N-terminal peptide.
***Sodium-glucose cotransporter 2 (SGLT-2) inhibitors***		
Correale M et al., 2021 [252]	Cross-sectional study; 22 patients with chronic HF and type 2 diabetes mellitus vs. n = 23 controls treated with other antidiabetic drugs	SGLT2 i administration improved endothelial function and arterial stiffness.
Sezai A et al., 2019 [253]	Prospective cohort study; 35 Japanese patients with chronic HF; administration of canagliflozin for 12 months.	Administration of canagliflozin decreased fat content at 12 months. Moreover, significant decrease in natriuretic peptides, improvement of renal function, FMD, E/e’ and oxidized LDL levels were reported after canagliflozin administration.
***Sacubitril/Valsartan***		
Li BH et al., 2021 [245]	RCT; 80 patients with HFrEF; randomized to observation group (n = 40, sacubitril/valsartan plus conventional treatment) vs. control group (n = 40, perindopril plus conventional treatment) for 12 weeks.	Sacubitril valsartan improves endothelial function while increasing cardiac function in HFrEF patients.
Bunsawat K et al., 2021 [254]	Prospective cohort study; 11 patients with HFrEF under optimal treatment; administration of sacubitril-valsartan for 3 months.	Administration of sacubitril/valsartan improved FMD at 1 month and at 2-3 months. Moreover, decreased levels of TNF-α and IL-18 were reported.
Du H et al., 2022 [255]	RCT; 60 patients with chronic HF and hypertension; randomly divided into observation group (n = 30, sacubitril/valsartan) and control group (n = 30, valsartan) for 6 months.	Sacubitril/valsartan subjects reported improvement in endothelium-dependent vasodilation, serum NO, and decreased ET-1 levels.
Nathaniel S et al., 2022 [246]	Case-control study; 20 HFrEF patients (n = 10 on sacubitril/valsartan vs. n = 10 on conventional treatment with ACEi/ARBs) for 12 weeks.	Sacubitril/valsartan decreased PWV and improved FMD.
Amore L et al., 2022 [244]	Prospective cohort study; 15 patients with dilated cardiomyopathy and reduced LVEF; administration of sacubitril/valsartan for 6 months.	Administration of sacubitril/valsartan shows an increase in reactive hyperemia index and AIx six months after first administration.
***Endothelin receptor antagonists (ERAs)***		
Karavolias GK et al., 2010 [256]	Cross-sectional study; 16 patients with moderate-severe idiopathic PAH under conventional treatment; administration of bosentan (62.5 mg twice daily for 1 month followed by 125 mg twice daily for 11 months).	Bosentan therapy modifies endothelial cell activation by down-regulating the levels of ICAM-1 at 2 months.
Iannone F et al., 2008 [257]	Cross-sectional study; 35 patients with systemic sclerosis (n = 10 with isolated PAH) vs. n = 25 healthy subjects; administration of bosentan in patients with isolated PAH.	Administration of bosentan for 12 months down-regulated endothelial activation and reduced ICAM-1, VCAM-1, P-selectin, PECAM-1, CD3 LFA-1 T, and CD3-L-selectin T-cell levels.
Sfikakis PP et al., 2007 [258]	RCT; Cross-sectional study, 12 patients with systemic sclerosis/PAH who received bosentan for 4 weeks vs. n = 12 patients without bosentan.	Small doses of bosentan improve endothelial function without affecting hemodynamic parameters or endothelial activation-related processes.

ADMA: asymmetric dimethylarginine; ACEi: Angiotensin-converting enzyme inhibitor; Alx: Augmentation index; ARBs: Angiotensin Receptor Inhibitors; BNP: Brain natriuretic peptide; EID: endothelial independent dilation; EPCs: endothelial progenitor cells; ERAs: Endothelin receptor antagonists; ET: Exercise training; ET-1: Endothelin-1; FMD: flow-mediated dilatation; HFrEF: heart failure with reduced ejection fraction; HFmrEF: heart failure with mildly reduced ejection fraction; HFpEF: heart failure with preserved ejection fraction; ICD: Implantable cardioverter defibrillator; IL: interleukin; IVUS: Intravascular ultrasound; LFA-1: Lymphocyte function-associated antigen-1; LVEF: left ventricular ejection fraction; LVFS: left ventricular short-axis shortening rate; LVEDD: left ventricular end-diastolic diameter; LVESD: left ventricular end-systolic diameter; MRA: Mineralocorticoid Receptor Antagonists; MMP: matrix metalloproteinase; MPO: myeloperoxidase; NT-proBNP: N-terminal pro-brain natriuretic peptide; oxLDL: oxidized low-density lipoprotein; PAH: Pulmonary arterial hypertension; PAP: Pulmonary artery pressure; PECAM-1: platelet/endothelial cell adhesion molecule; PerOx: total lipid peroxides; PVR: pulmonary vascular resistance; PWV: Pulse wave velocity; RCT: Randomized controlled trial; RHI: reactive hyperemia index; RH-PAT; reactive hyperemia-peripheral arterial tonometry; SGLT2 i: Sodium-glucose-cotransporter-type-2 inhibitors; sVCAM-1: soluble vascular cell adhesion molecule; sICAM-1: soluble intercellular adhesion molecule; SDF-1: stromal-derived factor 1; SMD: Standardized mean difference; TNF-α: tumor necrosis factor alpha; VEGF: vascular endothelial growth factor; vWF: von Willebrand factor.

**Table 2:** Ongoing clinical trials evaluating the therapeutic modalities against endothelial dysfunction in heart failure.

Clinical Trial Identifier/Official Title	Study Design/Estimated Enrollment/Inclusion Criteria	Primary Outcome Measures	Secondary Outcome Measures
NCT04539093 “Assessment of Endothelial Function in Patients with Advanced Heart Failure Requiring Mechanical Circulatory Support”	Prospective cohort; n = 20 patients with end-stage HF scheduled for LVAD implantation	Evaluation of endothelial function (blood NO levels, FMD) Evaluation of microvascular function (contrast-enhanced ultrasound of the peripheral skeletal muscle of lower extremities)	Functional outcomes: Quality of Life (KCCQ); Mobility (6 MWT); Handgrip; Lower extremity strength; Ventilation and gas exchange (CPET)
NCT05230732 “Neuromodulation of Inflammation and Endothelial Function to Treat Elderly Patients With Systolic Heart Failure”	Prospective, randomized, double-blind study; n = 158 patients with systolic HF and EF < 40%; use of Low-level Tragus stimulation- vs. sham treatment for 1 h daily/12 weeks	6 MWT	Quality of life (Minnesota living with heart failure questionnaire) FMD HRV Inflammatory cytokines
NCT02997462 “Monocyte Phenotypic Changes in Heart Failure”	Prospective cohort; n = 60 patients with HF admitted to the ICU or HF service vs. healthy, age-matched controls	Change in IL-6 levels between hospital admission and discharge	Evaluation of cell surface markers, cytokines, monocyte-gene expression, microRNA, monocyte/macrophage morphology, markers of oxidative stress and inflammation at admission, discharge, and at first post-discharge appointment
NCT04323371 “Cardiogenic Shock Integrated PHenotyping for Event Reduction”	Prospective observational study; n = 26 patients with acute decompensated HF complicated by cardiogenic shock	Markers of inflammation, endothelial permeability, endothelial glycocalyx perturbation, and metabolomics profile	
NCT05498584 “Targeting LOXL2 and Cardiac Fibrosis for Post-acute Heart Failure Treatment- A Prospective Study”	Prospective cohort study; n = 126 patients with HF (EF ≤ 40%) admitted for worsening HF in 3 years; administration of a cardiac rehabilitation program according to cardiopulmonary exercise test	All-cause mortality	HF readmission FMD (change from baseline to 6 months) KCCQ (change from baseline to 6 and 12 months)

6 MWT: six-minute walk test; CPET: Cardiopulmonary Exercise Test; EF: Ejection fraction; HF: Heart failure; HGS: Handgrip strength; HRV: Heart rate variability; FMD: Flow mediated dilatation; ICU: Intensive care unit; KCCQ: Kansas City Cardiomyopathy Questionnaire; LVAD: left ventricular assist devices; NO: Nitric oxide.

## Data Availability

Data sharing not applicable. No new data were created or analyzed in this study. Data sharing is not applicable to this article.
